# Shared genetic risk across different presentations of gene test–negative idiopathic nephrotic syndrome

**DOI:** 10.1007/s00467-022-05789-7

**Published:** 2022-11-10

**Authors:** Mallory L. Downie, Sanjana Gupta, Melanie M. Y. Chan, Omid Sadeghi-Alavijeh, Jingjing Cao, Rulan S. Parekh, Carmen Bugarin Diz, Agnieszka Bierzynska, Adam P. Levine, Ruth J. Pepper, Horia Stanescu, Moin A. Saleem, Robert Kleta, Detlef Bockenhauer, Ania B. Koziell, Daniel P. Gale

**Affiliations:** 1 Department of Renal Medicine, University College London, 1st Floor, Royal Free Hospital, Rowland Hill Street, London, NW3 2PF UK; 2 Paediatric Nephrology, Great Ormond Street Hospital for Children NHS Foundation Trust, London, UK; 3 Department of Medicine, Women’s College Hospital, Toronto, Canada; 4 Department of Pediatrics, Division of Nephrology, The Hospital for Sick Children, Toronto, Canada; 5 Department of Paediatric Nephrology, Evelina London and Faculty of Life Sciences, King’s College London, London, UK; 6 Bristol Renal, Translational Health Sciences, Bristol Medical School, University of Bristol, Bristol, UK; 7 Research Department of Pathology, University College London, London, UK

**Keywords:** Steroid-sensitive nephrotic syndrome, Steroid-resistant nephrotic syndrome, Paediatrics, Genetic risk score, Minimal change disease, Focal segmental glomerulosclerosis, Monogenic

## Abstract

**Background:**

Idiop athic nephrotic syndrome (INS) is classified in children according to response to initial corticosteroid therapy into steroid-sensitive (SSNS) and steroid-resistant nephrotic syndrome (SRNS), and in adults according to histology into minimal change disease (MCD) and focal segmental glomerulosclerosis (FSGS). However, there is well-recognised phenotypic overlap between these entities. Genome-wide association studies (GWAS) have shown a strong association between SSNS and variation at HLA, suggesting an underlying immunological basis. We sought to determine whether a risk score generated from genetic variants associated with SSNS could be used to gain insight into the pathophysiology of INS presenting in other ways.

**Methods:**

We developed an SSNS genetic risk score (SSNS-GRS) from the five variants independently associated with childhood SSNS in a previous European GWAS. We quantified SSNS-GRS in independent cohorts of European individuals with childhood SSNS, non-monogenic SRNS, MCD, and FSGS, and contrasted them with SSNS-GRS quantified in individuals with monogenic SRNS, membranous nephropathy (a different immune-mediated disease-causing nephrotic syndrome), and healthy controls.

**Results:**

The SSNS-GRS was significantly elevated in cohorts with SSNS, non-monogenic SRNS, MCD, and FSGS compared to healthy participants and those with membranous nephropathy. The SSNS-GRS in all cohorts with non-monogenic INS were also significantly elevated compared to those with monogenic SRNS.

**Conclusions:**

The shared genetic risk factors among patients with different presentations of INS strongly suggests a shared autoimmune pathogenesis when monogenic causes are excluded. Use of the SSNS-GRS, in addition to testing for monogenic causes, may help to classify patients presenting with INS.

**Graphical abstract:**

**Figure:**
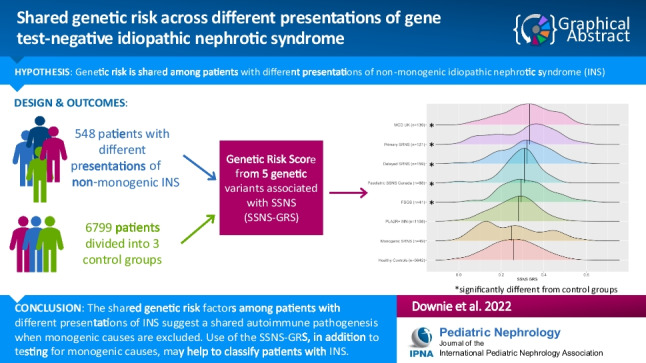
A higher resolution version of the Graphical abstract is available as Supplementary information

**Supplementary Information:**

The online version contains supplementary material available at 10.1007/s00467-022-05789-7.

## Introduction

Idiopathic nephrotic syndrome (INS) is the most common childhood kidney disease worldwide [1]. Children with INS are subclassified according to their response to first-line treatment with corticosteroids into steroid-sensitive nephrotic syndrome (SSNS, responsive to corticosteroids within 4 weeks) and steroid-resistant nephrotic syndrome (SRNS, unresponsive to corticosteroids after 4 weeks) [2]. Traditionally, SSNS and SRNS are considered as separate disease entities, with SRNS carrying a worse prognosis. Associations with kidney biopsy findings have also been used to distinguish the two entities, with SSNS biopsies characteristically demonstrating minimal change disease (MCD) histopathology and biopsies in SRNS typically showing focal segmental glomerulosclerosis (FSGS) [3–5].

Currently, testing for monogenic disease is recommended in patients with SRNS because pathogenic variants of podocyte-expressed genes are identifiable in 5–30% of such cases and these patients typically do not respond to intensified immunosuppression [6]. Where causative variants are not detected, however, there is evidence to suggest that SSNS and SRNS represent a spectrum of a single disease [7, 8], and a trial of alternative immunosuppressive therapy is often initiated. Typically, around 70% of children with non-monogenic SRNS go on to exhibit partial or complete remission if treated with immunosuppressants such as calcineurin inhibitors [9], and there is growing evidence that targeting B cells with anti-CD20 monoclonal antibodies is effective in at least half of the remaining patients [10] despite initial corticosteroid resistance [11]. Furthermore, there are patients who have initial response to corticosteroid therapy who develop secondary steroid resistance and some may show histopathological evolution from MCD to FSGS on kidney biopsy [7]. There are also studies of familial INS where individuals within the same pedigree can have differing nephrotic syndrome phenotypes and outcomes—such as MCD and SSNS in one sibling and FSGS and poor response to medication in another [12]. These clinical observations highlight the overlapping phenotype of SSNS, non-monogenic (i.e. gene test–negative) SRNS, MCD, and FSGS, suggesting a shared underlying pathophysiology.

Recent genome-wide association studies (GWAS) have demonstrated the polygenic nature of SSNS, identifying associated alleles in *HLA-DQA1/DRB1* and *HLA-DQB1* as well as several non-HLA loci [13–16]. These findings support the long-recognised clinical observations that SSNS is an immune-mediated disease. Genotyping these variants and weighting them by their observed effect on risk to create an SSNS genetic risk score (SSNS-GRS) allow estimation of an individual’s genetic risk for developing SSNS in a given population. Genetic risk scores can be aggregated within clinically defined groups to detect and allow comparison of genetic risk for the disease in a variety of clinical settings [17–19]. To further understand the observed clinical overlap between SSNS, non-monogenic SRNS, MCD, and idiopathic FSGS in Europeans, we sought to determine whether the known genetic risk factors for these disorders are shared and if they can be used to discriminate from monogenic causes of disease.

## Methods

### Study populations

This study was approved by the Institute of Child Health/Great Ormond Street Hospital Research Ethics Committee (ref 05/Q0508/6). The study cohorts analysed were of European ancestry and included paediatric SSNS (*n* = 88, none of whom were included in the original GWAS from which the SSNS-GRS was derived); adult biopsy-proven MCD (*n* = 139); paediatric monogenic SRNS (where a clinically reportable monogenic cause for proteinuric kidney disease had been identified on genetic testing by the UK National genetic testing service [20], *n* = 49); paediatric non-monogenic SRNS (with no clinically reportable monogenic variant identified on genetic testing), divided as either primary (unresponsiveness to corticosteroid therapy after 4 weeks of treatment, *n* = 121) or delayed (initial response to corticosteroids followed by resistance at some later time, *n* = 159); adult biopsy-proven idiopathic FSGS (*n* = 41); adult kidney disease controls with biopsy-proven anti-PLA2R positive membranous nephropathy (*n* = 1108); and healthy controls (*n* = 5642). INS diagnoses were defined according to KDIGO guidelines [2]. Individuals of European ancestry were identified by principal component analysis (PCA) using a subset of common variants in linkage equilibrium. Details of the study populations are as follows (also displayed in Supplementary Table S1):

1. Paediatric SSNS Canada: 88 children (<18 years of age) of European ancestry recruited from the INSIGHT study [21] (Toronto, Canada) genotyped on Infinium Global Diversity Array-8 BeadChip and imputed using the TOPMed imputation reference panel [22].
2. MCD UK: 139 British adults (>18 years of age) with biopsy-proven MCD who participated in the MRC/KRUK National DNA Bank for Glomerulonephritis [23] and had genotyping performed on an Infinium Multi-Ethnic Global BeadChip.
3. Non-monogenic (Primary or Delayed) SRNS: 21 children (<18 years of age) with primary and 137 with delayed SRNS participating in the NIHR BioResource Rare Disease Study (BRIDGE) [24], along with 100 children (<18 years of age) with primary and 22 with delayed SRNS recruited from the University of Bristol. Data from BRIDGE consisted of whole-genome sequencing and we included individuals of European genetic ancestry only [25]. This cohort was depleted for monogenic causes of disease as recruitment to BRIDGE specifically excluded individuals with causal variants in podocyte genes known to be associated with monogenic SRNS previously detected by whole exome screening at King’s College London/Guy’s and St Thomas’ NHS Foundation Trust, and cases found to have a clinically reportable variant identified on application of a virtual panel comprising 57 diagnostic grade genes to the whole-genome sequencing data were excluded [24]. Any cases of SRNS secondary to systemic disease/obesity, or membranous/IgA nephropathy were also excluded. Patients from the University of Bristol were of self-reported European ancestry and were genotyped for the five target SNPs using an allele-specific polymerase-chain-reaction assay (LGC Group). This cohort included patients who did not have any clinically reportable variants on a sequencing panel of 53 genes associated with INS [20].
4. FSGS: 41 adults (>18 years of age) of European ancestry with biopsy-proven FSGS recruited from the Royal Free Hospital, London, UK (13 patients) and from the 100,000 Genomes Project [26] (28 patients). Patients from the Royal Free Hospital were all of self-reported European ancestry and were genotyped for the five target SNPs using an allele-specific polymerase-chain-reaction assay (LGC Group). Patients from the Royal Free Hospital did not have clinical genetic testing performed. Individuals from the 100,000 Genomes Project had whole-genome sequencing performed. Individuals with a known monogenic cause identified by the 100,000 Genomes Project expert, crowd-sourced review and internal curation process, facilitated by the PanelApp software [27], or other underlying disease were not included in this group.
5. Monogenic SRNS: 49 children (<18 years of age) of self-reported European ancestry in whom a clinically reportable variant in a gene associated with monogenic INS had been identified on genetic testing [20] were recruited from the University of Bristol and Guy’s and St Thomas’ NHS Foundation Trust. Individuals were genotyped for the five target SNPs using an allele-specific polymerase-chain-reaction assay (LGC Group).
6. Anti-PLA2R positive Membranous Nephropathy: Locally available data from 1108 adults (>18 years of age) of European ancestry with anti-PLA2R antibody positive membranous nephropathy (MN) [28]. Individuals were genotyped on an Infinium Multi-Ethnic Global BeadChip and imputed using the 1000 Genomes Reference Panel [29].
7. Healthy Controls: A cohort of healthy European individuals (*n* = 5642, children and adults) were used as controls, as previously detailed [13]. In brief, this included genotype data from individuals of European ancestry available in the European Genome Archive [30, 31], Illumina European ethnicity cohort [32], and the Wellcome Trust Case Control Consortium (WTCCC) [33]. Genotypes were imputed using the 1000 Genomes Reference Panel [29].

### Selection of variants and identification of individuals with European genetic ancestry

Where genome-wide or whole-genome data were available, a subset of 30,000–100,000 high-quality common variants not in linkage disequilibrium (LD) with each other was extracted from each genotyping/sequencing dataset for ancestry and relatedness estimation. For whole-genome sequencing datasets, selected variants were biallelic, and had GQ (genotype quality) >20, DP (read depth) >10, and OPR (overall pass rate) >0.8. In imputed datasets, selected variants were biallelic, and had call rate >95% and imputation score (Rsq) of >0.8. A kinship matrix was computed using KING [34], which was then used to identify the maximal set of unrelated individuals. The ancestry of samples included in the study was ascertained by calculating principal components (PC) in plink using unrelated 1000 Genomes [29] individuals and projecting the study genotypes onto this vector space. A multivariate model was then used to classify each subject as being non-Finnish European, Finnish, African, South Asian, and East Asian based on the 1000 Genomes data. Individuals of European ancestry were then selected. Completion of this quality control left 88 SSNS, 139 MCD, 280 SRNS, 41 FSGS, 49 monogenic INS, 1108 MN, and 5642 healthy control individuals.

### Calculation of the genetic risk score

**Fig. 1:**
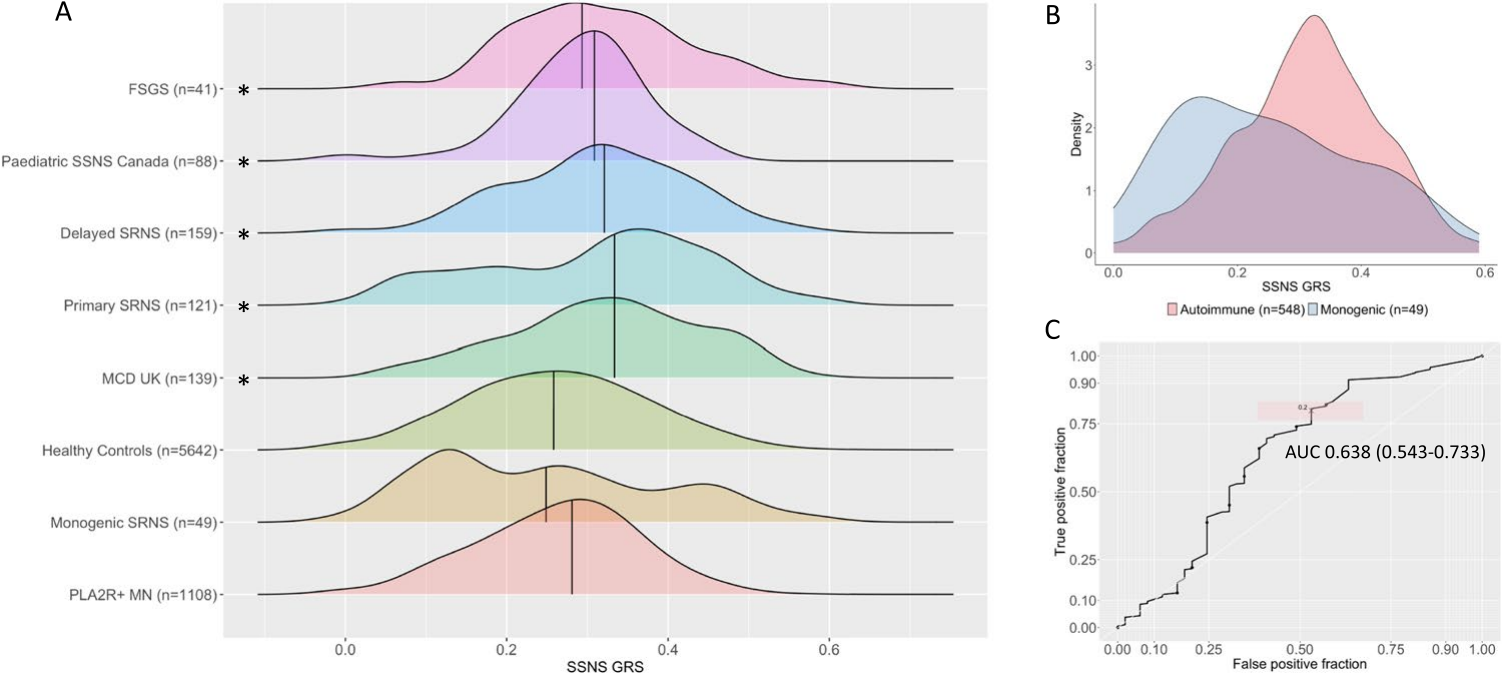
Genetic Risk Scores in SSNS, SRNS, MCD, and FSGS are elevated compared with control cohorts and monogenic INS. **A** Distribution of genetic risk scores in non-monogenic and monogenic INS compared to healthy controls. The *x-*axis represents SSNS-GRS. Median values for each group are represented by vertical lines with distribution of SSNS-GRS displayed through density plots for each cohort. Asterisks indicate *p* <0.007 using the Kruskal–Wallis test with correction for planned comparisons to healthy controls. **B** Density of SSNS-GRS in individuals with monogenic versus autoimmune INS. **C** ROC curve for monogenic versus autoimmune INS. SSNS, steroid-sensitive nephrotic syndrome; MCD, minimal change disease; SRNS, steroid-resistant nephrotic syndrome; FSGS, focal segmental glomerulosclerosis; INS, idiopathic nephrotic syndrome; GRS, genetic risk score; ROC, receiver operating characteristic

SSNS-GRSs were calculated using the odds ratios at each of the five independent risk loci identified from our previously reported European SSNS GWAS (see Table 1) [13]. Where specific variants were not available in the dataset, the highest quality SNP in tightest linkage disequilibrium (LD) was selected (*R*^2^ > 0.8) (see Supplementary Table S2). In cohorts where alternate SNPs were used, GRS-SSNS was calculated in the control cohort using identical SNPs and verified to be the same as reported in Fig. 1A. SSNS-GRS was determined by the sum of the natural logarithm (ln) of the odds ratio multiplied by the number of risk alleles at each locus (0, 1, or 2), divided by the total number of possible alleles [35, 36]. At protective loci, values were computed using the natural logarithm of the inverse odds multiplied by the number of non-protective alleles.

**Table 1:** Independent genetic risk loci determined from European SSNS GWAS [13]

Marker	Gene	Test allele	Odds ratio	*p* value
rs9273542	*HLA-DQB1*	T	3.39	1.59 × 10^−43^
rs2858317	*HLA-DQB1*	C	0.37	4.28 × 10^−31^
rs3828799	*HLA-DQB1*	C	1.81	2.40 × 10^−8^
rs2637678	*CALHM6*	C	0.51	1.27 × 10^−17^
rs10518133	*PARM1*	A	1.96	2.50 × 10^−8^

Risk loci were determined from a previously published GWAS in European children with SSNS (422 cases and 5642 healthy controls [13])

*SSNS* steroid-sensitive nephrotic syndrome, *GWAS* genome-wide association study

Continuous data are reported as medians with interquartile range. SSNS-GRSs were compared using the Kruskal–Wallis test due to non-parametric distribution of the data, with correction for seven planned comparisons using a *p*-value threshold of *p* <0.007 (0.05/7). The SSNS-GRS with the best discriminative capacity between non-monogenic and monogenic INS was determined based on the maximal area under the receiver operating characteristic curve (AUC). Statistical tests and data visualisation were performed in R v4.1.1.

## Results

Median (and interquartile range) SSNS-GRS for each of the study populations were as follows: paediatric SSNS 0.31 (0.26–0.33), MCD 0.33 (0.25–0.41), primary non-monogenic SRNS 0.33 (0.19–0.40), delayed non-monogenic SRNS 0.32 (0.24–0.38), and idiopathic FSGS 0.29 (0.23–0.38). The differences between all these INS groups were not statistically significant (*p* > 0.14). Median SSNS-GRS among patients with monogenic SRNS was 0.25 (0.13–0.33) and among healthy European patients was 0.26 (0.17–0.33), significantly lower than all of the INS groups (*p* < 0.007) and similar to that of a cohort of 1108 European patients with biopsy-proven membranous nephropathy, among whom the SSNS-GRS was 0.28 (0.19–0.33) (see Fig. 1A and Supplementary Table S3). These results therefore showed that, compared with healthy individuals, all the groups with non-monogenic INS (whether presenting with SSNS, non-monogenic SRNS, MCD or FSGS, in childhood or adulthood) exhibited similarly elevated SSNS-GRS. In contrast, among individuals with monogenic SRNS (or individuals with membranous nephropathy), the SSNS-GRS was not elevated compared with healthy patients.

Receiver operating characteristic (ROC) analysis comparing SSNS-GRS in non-monogenic (*n* = 548) and monogenic (*n* = 49) INS demonstrated the best discriminative SSNS-GRS value to be 0.2 with AUC of 0.638 (0.543–0.733) (see Fig. 1B, C). This means that individuals with SSNS-GRS <0.2 were more likely to have monogenic INS, and vice versa.

## Discussion

Phenotypic variability of nephrotic syndrome most likely represents a spectrum of disease along a continuum. In this study, we present a novel genetic risk score developed from the largest European GWAS in patients with SSNS to date [13]. The overlap in SSNS-GRS between groups of European-ancestry patients with SSNS and other non-monogenic presentations of INS demonstrates that genetic risk factors are shared by patients with these presentations. This implies that shared pathogenic mechanisms are operating across these conditions. While future genetic studies may identify additional genetic factors that distinguish the groups, the possibility exists that these forms of INS are all differing presentations of the same underlying disorder, an acquired podocytopathy. The aetiology is almost certainly autoimmune in nature, based on the predominance of variants in the HLA region that determine the genetic risk. This contention is further supported by the recent identification of anti-nephrin antibodies in a subset of patients with MCD [37] and, of course, by the clinical response of most of these patients to immunosuppressive treatments. The SSNS-GRS is also able to discriminate between non-monogenic INS and membranous nephropathy (another immune-mediated disease manifesting in nephrotic syndrome), demonstrating that an elevated SSNS-GRS is not observed in another HLA-driven immune-mediated disease.

The similarity of SSNS-GRS between non-monogenic SRNS (both primary and delayed) and SSNS suggests that, for the purposes of understanding the disease pathogenesis and selecting treatments, those patients presenting with SRNS in whom an underlying monogenic disorder is not detected are likely to have an autoimmune podocytopathy, just as those presenting with SSNS. This is further supported by our findings that individuals with (currently recognised) monogenic SRNS do not exhibit an elevated SSNS-GRS. Thus, it would be possible to replace the disease labels SSNS and SRNS by the terms presumptive autoimmune podocytopathy (referring to almost all steroid-sensitive and most initially steroid-resistant cases) and monogenic podocytopathy (referring to the minority of steroid-resistant cases in whom there is evidence of a monogenic cause), with the disorders differentiated by genetic testing. This distinction between a monogenic podocytopathy (due to pathogenic variant(s) in a gene important for podocyte function) or autoimmune podocytopathy (due to acquired immune-mediated podocyte damage) is consistent with the clinical experience that most patients with non-monogenic disease respond to immunosuppressive treatments and, if they do not, have a high risk of disease recurrence post-transplantation (which again typically responds to intensified immunosuppression) [38].

We also aimed to address the question of whether use of the SSNS-GRS has the potential to provide a clinically useful prior probability of an underlying monogenic disorder more rapidly and with less harm than current practice, which is an empirical trial of high-dose corticosteroid treatment. However, ROC analysis indicated that an SSNS-GRS of 0.2 was the optimum value to discriminate between cohorts with autoimmune and monogenic INS, which was below the median SSNS-GRS value in both groups, indicating a substantial false classification rate to diagnose patients using the SSNS-GRS alone. This is consistent with the AUC of only 0.638 (0.543–0.733), which implies that this SSNS-GRS is not reliable enough to distinguish disease aetiology in an individual patient. However, if other biomarkers (such as serological tests) become available to clinicians in the future then utility of an SSNS-GRS might be re-examined.

The similarity in SSNS-GRS between European adults with MCD or idiopathic FSGS and the paediatric INS groups suggests that adult-onset INS can share the same underlying aetiology which can be associated with either MCD or FSGS on histology. A shared pathophysiology underlying idiopathic FSGS and the other INS groups is supported by the following observations: first, relapsing MCD can, over time, develop into FSGS [7]; second, where INS recurs following transplantation, early allograft biopsy shows extensive podocyte foot process effacement with no light microscopic glomerular abnormalities evolving into established FSGS in most cases over the course of a year [7]; third, that FSGS is the histological abnormality most likely to be detected in patients presenting with SRNS [39].

Our study has limitations. First, it only examines patients of European ancestry. Further studies are needed to confirm whether a genetic overlap between SSNS and SRNS also applies to other groups, such as African populations where *APOL1* risk variants may have an important impact. Second, genome-wide data was not available for all our cohorts which limited the use of polygenic risk score programs that incorporate information from large numbers of non-genome-wide significant markers. Lastly, while the SSNS-GRS distinguished between monogenic and non-monogenic INS cohorts in this study, knowing the SSNS-GRS in an individual patient at this stage cannot directly inform the management of that patient because of the substantial overlap between the various groups (Fig. 1). Future identification of more variants associated with autoimmune INS might help to refine the risk score and thereby improve its ability to distinguish monogenic and non-monogenic disease.

## Conclusions

Shared genetic risk factors are present in individuals presenting with SSNS, MCD, FSGS, and non-monogenic SRNS, suggesting that these entities share a common pathophysiological mechanism. In view of the strong HLA association, the shared aetiology is almost certainly autoimmune in nature. Furthermore, SSNS-GRSs in individuals with monogenic INS were not elevated compared to controls. Accordingly, we propose classification of idiopathic nephrotic syndrome into monogenic versus autoimmune podocytopathy rather than by response to empirical corticosteroids or by biopsy appearances. In the future, use of an SSNS-GRS may aid in the classification of individual patients with INS, in addition to testing for monogenic causes.

## Supplementary Information

Below is the link to the electronic supplementary material.

Graphical Abstract (PPTX 185 KB)

Supplementary file2 (DOCX 24.7 KB)

## Declarations

### Disclaimer

The views expressed are those of the authors and not necessarily those of the NHS, the NIHR, or the Department of Health and Social Care.
