# Supplementary material for: Shared genetic risk across different presentations of gene test–negative idiopathic nephrotic syndrome
Source: Pediatr Nephrol. 2022 Nov 10;38(6):1793–800. doi: 10.1007/s00467-022-05789-7 (PMC10154254; doi:10.1007/s00467-022-05789-7)
Supplement: Supplementary file 1 — Graphical Abstract (PPTX 185 KB) [file 467_2022_5789_MOESM1_ESM.pptx]

## Slide 1
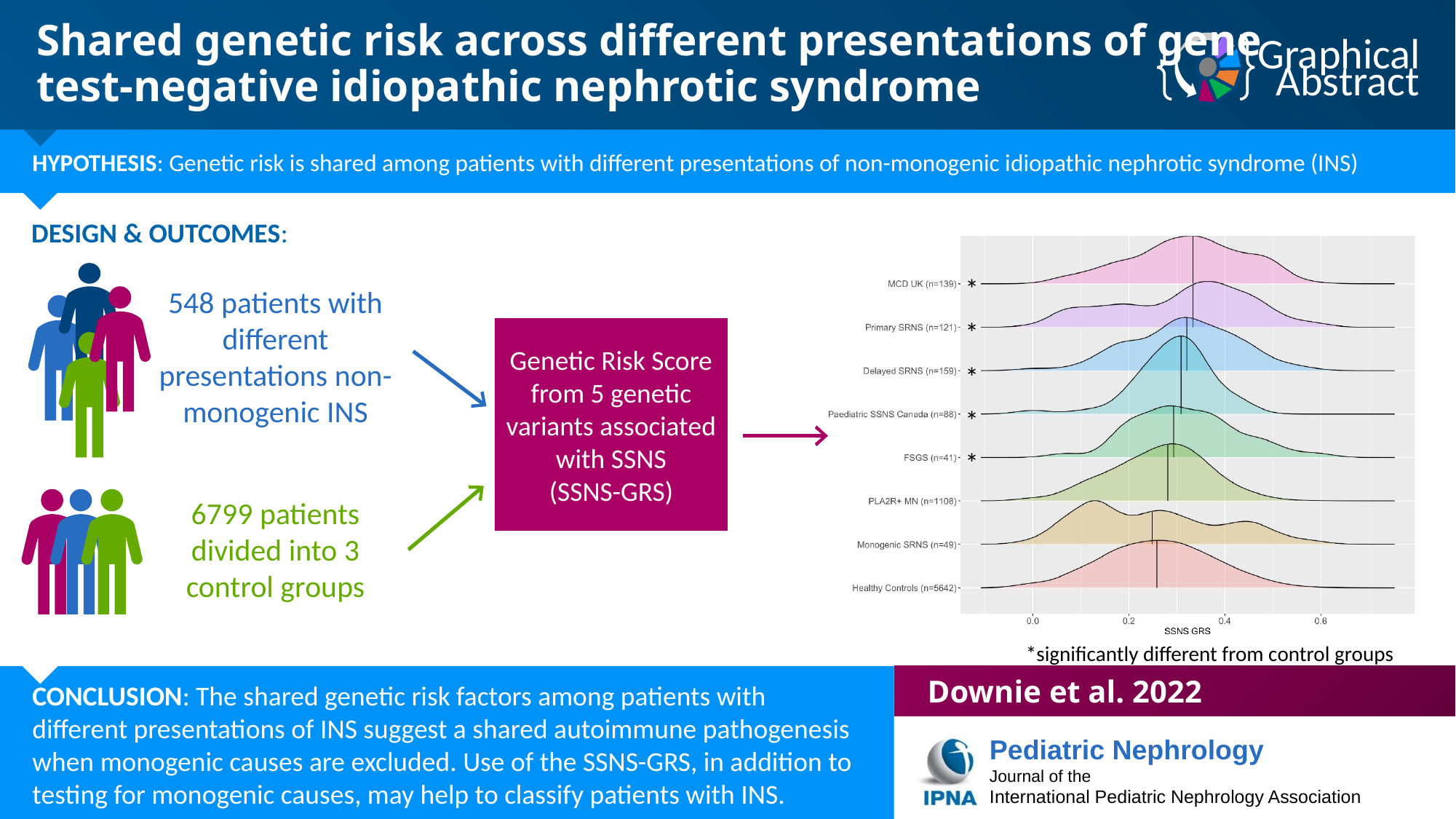

Shared genetic risk across different presentations of gene
test-negative idiopathic nephrotic syndrome
HYPOTHESIS: Genetic risk is shared among patients with different presentations of non-monogenic idiopathic nephrotic syndrome (INS)
DESIGN & OUTCOMES:
*
548 patients with different presentations non-monogenic INS
*
Genetic Risk Score from 5 genetic variants associated with SSNS
(SSNS-GRS)
*
*
*
6799 patients divided into 3 control groups
*significantly different from control groups
Downie et al. 2022
CONCLUSION: The shared genetic risk factors among patients with different presentations of INS suggest a shared autoimmune pathogenesis when monogenic causes are excluded. Use of the SSNS-GRS, in addition to testing for monogenic causes, may help to classify patients with INS.
