# Supplementary material for: Shared genetic risk across different presentations of gene test–negative idiopathic nephrotic syndrome
Source: Pediatr Nephrol. 2022 Nov 10;38(6):1793–800. doi: 10.1007/s00467-022-05789-7 (PMC10154254; doi:10.1007/s00467-022-05789-7)
Supplement: Supplementary file 2 — Supplementary file2 (DOCX 24.7 KB) [file 467_2022_5789_MOESM2_ESM.docx]

**Supplementary material**

| **Cohort** | **Number of participants** | **Age of participants** | **Genetic dataset** | **Ancestry determination** |
| --- | --- | --- | --- | --- |
| Paediatric SSNS Canada | 88 | <18 years | Genotyped and imputed | PCA |
| MCD UK | 139 | >18 years | Genotyped | PCA |
| Non-monogenic Primary SRNS | |  |  |  |
| BRIDGE | 21 | <18 years | WGS | PCA |
| Bristol | 100 | <18 years | 5-SNP genotype | Self-report |
| Non-monogenic Delayed SRNS | |  |  |  |
| BRIDGE | 137 | <18 years | WGS | PCA |
| Bristol | 22 | <18 years | 5-SNP genotype | Self-report |
| FSGS |  |  |  |  |
| RFH | 13 | >18 years | 5-SNP genotype | Self-report |
| GEL | 28 | >18 years | WGS | PCA |
| Monogenic SRNS | 49 | <18 years | 5-SNP genotype | Self-report |
| PLA2R+ MN | 1108 | >18 years | Genotyped and imputed | PCA |
| Healthy controls | 5642 | Any | Genotyped and imputed | PCA |

**Table S1: SSNS-GRS cohort characteristics**

SSNS, steroid sensitive nephrotic syndrome; GRS, genetic risk score; MCD, minimal change disease; SRNS, steroid resistant nephrotic syndrome; BRIDGE, NIHR BioResource Rare Disease Study; Bristol, University of Bristol; FSGS, focal segmental glomerulosclerosis; RFH, Royal Free Hospital; GEL, Genomics England 100,000 Genomes Project; PLA2R+, phospholipase A2 receptor positive; MN, membranous nephropathy; SNP, single nucleotide polymorphism; WGS, whole genome sequencing; PCA, principal component analysis

|  | **Pediatric SSNS Canada** | **MCD UK** | **Non-monogenic SRNS**  **BRIDGE** | **FSGS RFH** | **FSGS GEL** | **Non-monogenic & monogenic SRNS**  **Bristol** | **PLA2R+ MN** | **Healthy controls** |
| --- | --- | --- | --- | --- | --- | --- | --- | --- |
| SNP in LD with rs9272542 *(HLA-DQB1)* | rs9274623 | rs3891176 | rs9272542 | rs9273529 | rs9272542 | rs9273529 | rs9272542 | rs9272542 |
| Test Allele | T | A | T | T | T | T | T | T |
| SNP in LD with rs2858317 *(HLA-DQB1)* | rs2858319 | rs4273729 | rs2856668 | rs2858317 | rs2858317 | rs2858317 | rs2858317 | rs2858317 |
| Test Allele | T | C | G | C | C | C | C | C |
| SNP in LD with rs3828799 *(HLA-DQB1)* | rs3828799 | rs28724252 | rs9274656 | rs3828800 | rs3828799 | rs3828800 | rs3828799 | rs3828799 |
| Test Allele | C | A | C | A | C | A | C | C |
| SNP in LD with rs2637678 *(CALHM6)* | rs2637678 | rs9384981 | rs2637678 | rs2637678 | rs2637678 | rs2637678 | rs2637678 | rs2637678 |
| Test Allele | C | C | C | C | C | C | C | C |
| SNP in LD with rs10518133 *(PARM1)* | rs10518133 | rs6824037 | rs10518133 | rs10518133 | rs10518133 | rs10518133 | rs10518133 | rs10518133 |
| Test Allele | A | T | A | A | A | A | A | A |

Table S2: Matched and proxy SNPs used to calculate the SSNS-GRS in study cohorts

Identical and proxy SNPs in LD with the European SSNS GWAS lead SNPs for the 5 independent SNPs for each cohort are listed here. These are the SNPs used to calculate the SSNS-GRS. Boxes highlighted in grey represent identical SNPs to the European SSNS GWAS used in the score. All proxy SNPs had R^2^≥0.8. LD, linkage disequilibrium; GRS, genetic risk score; SSNS, steroid sensitive nephrotic syndrome; MCD UK, minimal change disease United Kingdom; SRNS, steroid resistant nephrotic syndrome; BRIDGE, NIHR BioResource Rare Disease Study; Bristol, University of Bristol; FSGS RFH, focal segmental glomerulosclerosis Royal Free Hospital; GEL, Genomics England 100,000 Genomes Project; PLA2R+, phospholipase A2 receptor positive; MN, membranous nephropathy

|  | **Delayed SRNS** | **FSGS** | **Healthy Controls** | **MCD UK** | **Monogenic SRNS** | **Paediatric SSNS Canada** | **PLA2R+ MN** |
| --- | --- | --- | --- | --- | --- | --- | --- |
| **FSGS** | 0.446 | - | - | - | - | - | - |
| **Healthy Controls** | <0.001 | <0.001 | - | - | - | - | - |
| **MCD UK** | 0.254 | 0.382 | <0.001 | - | - | - | - |
| **Monogenic SRNS** | <0.001 | 0.002 | 0.192 | <0.001 | - | - | - |
| **Paediatric SSNS Canada** | 0.146 | 0.193 | 0.001 | 0.056 | 0.006 | - | - |
| **PLA2R+ MN** | <0.001 | 0.002 | 0.190 | <0.001 | 0.147 | 0.004 | - |
| **Primary SRNS** | 0.364 | 0.358 | <0.001 | 0.170 | <0.001 | 0.242 | <0.001 |

**Table S3: Test statistics for comparison of SSNS-GRS in study cohorts**

SSNS-GRS were compared using the Kruskal-Wallis test with correction for seven planned comparisons using a p-value threshold of p<0.007 (0.05/7). This table shows the p-values for the statistical comparison and demonstrates that compared with healthy individuals, all groups with non-monogenic idiopathic nephrotic syndrome (SSNS, MCD, non-monogenic primary SRNS, non-monogenic delayed SRNS, or FSGS) had significantly higher SSNS-GRS. In contrast, SSNS-GRS in cohorts with monogenic SRNS or PLA2R+ MN, SSNS-GRS was not significantly elevated. SSNS, steroid sensitive nephrotic syndrome; GRS, genetic risk score; MCD, minimal change disease; SRNS, steroid resistant nephrotic syndrome; FSGS, focal segmental glomerulosclerosis; PLA2R+, phospholipase A2 receptor positive; MN, membranous nephropathy
